# Refractive Profile and Angle of Deviation in Patients with Congenital Esotropia and Congenital Exotropia

**DOI:** 10.18502/jovr.v20.15066

**Published:** 2025-06-13

**Authors:** Masoud Khorrami-Nejad, Mohammad Reza Akbari, Ali Hassan Khaleel, Razieh Bahreini, Foroozan Narooie-Noori, Babak Masoomian

**Affiliations:** ^1^Optometry Department, School of Rehabilitation, Tehran University of Medical Sciences, Tehran, Iran; ^2^Department of Optical Techniques, Al-Mustaqbal University College, Hillah, Babylon, Iraq; ^3^Translational Ophthalmology Research Center, Farabi Eye Hospital, Tehran University of Medical Sciences, Tehran, Iran; ^4^Vision Science Department, Pacific University College of Optometry, Forest Grove, Oregon, USA; ^5^Wills Eye Hospital, Thomas Jefferson University, Philadelphia, PA, USA

**Keywords:** Angle of Deviation, Congenital Esotropia, Congenital Exotropia, Refractive Error

## Abstract

**Purpose:**

To compare refractive error and angle of deviation in patients with congenital esotropia (CET) and congenital exotropia (CXT).

**Methods:**

This retrospective study reviewed the medical documents of 246 patients with CET (*n* = 206) and CXT (*n* = 40) admitted to Farabi Eye Hospital, Iran, from 2012 to 2022. All patients were between 6 and 12 months old at the time of admission. Refractive error components and angles of deviation in these patients were recorded and analyzed.

**Results:**

In patients with CET, the mean sphere, cylinder, and spherical equivalent (SE) were 2.59 ± 2.28 diopters (D), –0.42 ± 0.57 D, and 2.38 ± 2.28 D, respectively. Also, the mean horizontal deviation at near was 45.5 ± 12.3 prism diopters (Δ) (range, 20–98 Δ). The most common range was 44–55 Δ (40%), followed by 33–44 Δ (28%) and 55–65 Δ (12%). On the other hand, the mean sphere, cylinder, and SE in patients with CXT were 1.88 ± 1.84 D, –0.39 ± 0.69 D, and 1.69 ± 1.74 D, respectively. Also, the mean horizontal deviation at near was 45.0 ± 17.1 Δ (range, 20–105 Δ). For the CXT group, deviation ranges of 33–44 Δ (37%), 44–55 Δ (32%), and 22–33 Δ (15%) were most prevalent. The mean sphere and SE were significantly higher in the CET group than in the CXT group (P = 0.010).

**Conclusion:**

This study found a distinct refractive profile and distribution of the angle of deviation in patients with CET versus CXT. Patients with CET demonstrated significantly greater hyperopia than those with CXT.

##  INTRODUCTION

As a common disorder in children, strabismus can lead to amblyopia and deficits in binocular vision if not treated properly.^[1,2]^ The two most common types of childhood strabismus are esotropia and exotropia.^[3,4]^ Congenital esotropia (CET) and congenital exotropia (CXT) manifest early in life, before six months of age, and are associated with large and constant angles of deviation ( > 30 prism diopters [ Δ ]).^[5,6]^ CET and CXT disrupt normal binocular vision development and often result in amblyopia, requiring early diagnosis and treatment to optimize visual outcomes.^[7,8,9]^

The underlying etiology of CET and CXT is not fully understood but is believed to involve anatomical, innervational, and hereditary factors.^[10]^ Compared to acquired esotropia and exotropia, CET and CXT tend to be more resistant to conservative treatments like glasses, occlusion therapy, and vision training.^[11]^ As a result, most children with CET and CXT require strabismus surgery to realign the eyes, ideally performed between 6 and 24 months of age.^[12]^ Numerous studies have examined the surgical outcomes of CET and CXT, with reoperation rates ranging from 27% to 77% due to under- or over-correction.^[13,14,15]^ Preoperative angle of deviation and refractive error are known prognostic factors for surgical success.^[16,17,18]^ However, few studies have directly compared the clinical characteristics of CET versus CXT, which could have implications for prognosis and management.

In particular, there is limited data on refractive error profiles in CET versus CXT. Most studies report that infants with CET have age-appropriate refractive errors, typically low to moderate hyperopia.^[3,19,20]^ Infants with CXT are thought to have a distribution of refractive errors similar to that of the general population.^[21]^ To date, no study has directly compared refractive error in CET and CXT. Additionally, while CET and CXT are both categorized as large-angle strabismus, few studies have quantitatively compared the angle of deviation. Clarifying these clinical characteristics in CET versus CXT could help identify optimal treatment strategies and prognostic factors.

The main aim of this study was to compare refractive error components and angle of deviation measurements between patients with CET and those with CXT.

##  METHODS

### Study Design

This retrospective, cross-sectional study reviewed medical records of patients who had undergone strabismus surgery between 2012 and 2022 at Farabi Eye Hospital, affiliated with Tehran University of Medical Sciences. All methods employed were in strict accordance with the Declaration of Helsinki. The Institutional Review Board and the Ethical Committee of Tehran University of Medical Sciences approved the study protocol (IR.TUMS.FNM.REC.1402.056). Following the university's policy and the approval of the Review Board, written consent was waived due to the study's retrospective design.

### Participants

The medical records of patients diagnosed with CET or CXT who had undergone strabismus surgery were included in the study. A total of 206 patients had CET and 40 patients had CXT. The inclusion criteria were initial diagnosis at age < 6 months, no prior strabismus surgery, no history of premature birth, no paralytic cause of strabismus, no neurological disorders, and no other systemic diseases. CET was defined as a non-accommodative, constant esodeviation developing in neurologically normal children within the first six months of age.^[22]^ CXT, on the other hand, was described as idiopathic and constant with onset within the first six months of life in infants with normal neurological development and no evidence of brain abnormalities.^[23,24,25]^ CXT was carefully differentiated from the normal, variable, small-angle exodeviation that can be observed in newborns without an underlying disorder.

### Data Collection

Data were extracted from preoperative medical records, including comprehensive eye examinations and strabismus measurements. The analyzed data comprised age, gender, cycloplegic refraction (spherical and cylindrical components), and angle of deviation.

### Measurements

Cycloplegic refraction was performed in all patients using cyclopentolate 1% and measured by HEINE BETA 200 Retinoscope (HEINE Optotechnik, Gilching, Germany). The spherical equivalent (SE) was calculated as the spherical power plus half the cylindrical power. Myopia was defined as an SE refractive error of –0.50 diopters (D) or less, while hyperopia was defined as a SE of +0.50 D or greater.^[26,27]^ Indirect ophthalmoscopy was performed for all patients as part of the preoperative evaluation process.

While assessing ocular alignment in infants is challenging, when possible, we used validated techniques such as the alternate prism cover test. However, since most patients were uncooperative, the angle of deviation was measured using the Krimsky test at near. This test determines the angle of constant deviation using hand-held prisms. The prism power required to center the corneal light reflex over the pupil corresponds to the angle of deviation. In cases with moderate and high hyperopic refractive error, a full cyclo refraction was initially prescribed if the cycloplegic refraction was ≥ 2.00 D.^[28]^ Patients whose esotropia resolved after the prescription of full cyclo refraction were excluded from the study. If the patients required glasses due to hyperopia, all measurements were conducted after they wore the corrective lenses.

Routinely, the ductions were carefully checked using the doll's head maneuver or by following the target for all patients. If the patient did not cooperate enough or was suspected of other abnormalities, the contralateral eye was patched for 30 minutes, and the ductions were rechecked. This test helps differentiate CET from Duane syndrome and sixth nerve palsy, especially when abduction is limited in congenital strabismus. In cases of CET, the duction test typically has normal results. However, the presence of limited abduction may indicate Duane retraction syndrome or sixth nerve palsy. Duane retraction syndrome, a congenital cranial dysinnervation disorder, is characterized by limited abduction, adduction, or both, along with a face turn and globe retraction on adduction. This is often caused by the co-contraction of the medial and lateral rectus muscles.^[29,30]^ Sixth nerve palsy, which results in limited abduction due to dysfunction of the abducens nerve, can lead to increased esotropia when looking toward the affected side. This condition can be either congenital or acquired from various causes.^[31]^

An experienced specialist in strabismus and pediatric ophthalmology measured the angle of deviation, and an expert optometrist measured refractive error.^[32,33]^ Both of these parameters were quantified and compared between the study groups.

In all cases, the first diagnosis of strabismus was made before the age of six months; however, the age at examination ranged from 6 to 12 months. Furthermore, all the cases underwent corrective strabismus surgery before the age of two years.

### Statistical Analysis

The collected data were analyzed using SPSS version 24 (IBM, Chicago, IL, USA). The normality tests were done using the Shapiro-Wilk test. The Mann-Whitney test was applied to explore significant differences between patients with CET and those with CXT. The Pearson correlation test was used to determine the correlation between the spherical refractive error and the angle of deviation. The distribution pattern of the angle of deviation was plotted in the two groups of patients using Microsoft Excel 2019 (Microsoft Corporation, Redmond, WA, USA). Box-and-Whisker plot for refractive error components and scatter plot of spherical refractive error against angle of deviation were drawn using MedCalc version 15.8.X86 (MedCalc Software bvba, Ostend, Belgium). A *P*-value < 0.05 was considered statistically significant.

**Table 1:** Comparison of refractive error components and horizontal deviation in patients with congenital esotropia and exotropia strabismus

			**Min to Max**	**Mean ± SD**	**MD ± SD**	***P*** -value*
Refractive error (D)	Sphere	Congenital esotropia (*n* = 206)	–9.50 to 12.50	2.59 ± 2.28	0.71 ± 3.63	0.010
		Congenital exotropia (*n* = 40)	–3.00 to 6.50	1.88 ± 1.84		
	Cylinder	Congenital esotropia (*n* = 206)	0.00 to –2.25	–0.42 ± 0.57	–0.03 ± 1.00	0.467
		Congenital exotropia (*n* = 40)	0.00 to –2.75	–0.39 ± 0.69		
	SE	Congenital esotropia (*n* = 206)	–10.00 to 12.00	2.38 ± 2.28	0.70 ± 3.64	0.010
		Congenital exotropia (*n* = 40)	–3.00 to 6.25	1.69 ± 1.74		
Angle of deviation (near, Δ )		Congenital esotropia (*n* = 206)	20.00 to 98.00	45.50 ± 12.30	0.63 ± 13.53	0.331
		Congenital exotropia (*n* = 40)	20.00 to 105.00	45.00 ± 17.10		
D, diopter; MD, mean difference; SD, standard deviation; SE, spherical equivalent						

**Figure 1:**
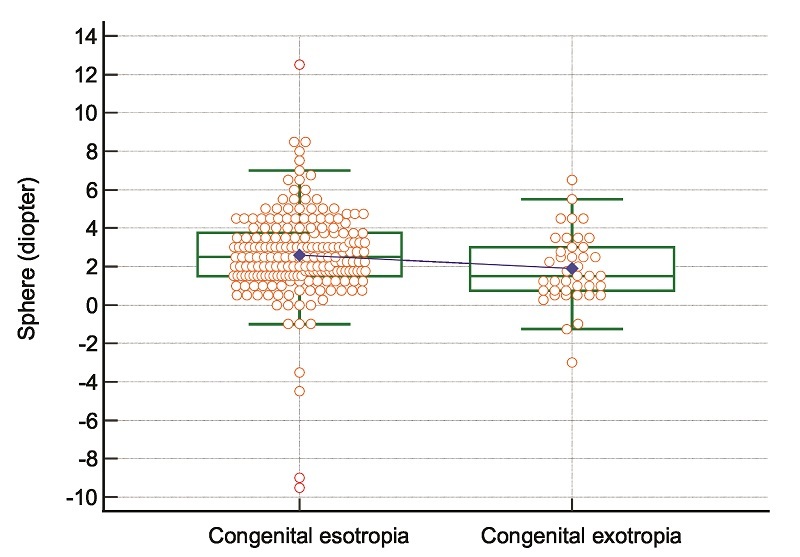
Box-and-Whisker plot for spherical refractive error in patients with congenital esotropia and congenital exotropia. The box is drawn from the 1 ${}^{st}$ to the 3 ${}^{\text{rd}}$ quartile of spherical refractive error. The horizontal line is drawn at the median of spherical refractive error, and the blue line connects the medians of the two groups. The interquartile range (IQR) is calculated as IQR = 3 ${}^{\text{rd}}$ quartile - 1 ${}^{st}$ quartile. And two imaginary lines, known as the upper and lower inner fences, are drawn at the 3 ${}^{\text{rd}}$ quartile ± 1.5 × IQR. The highest value of spherical refractive error just below the upper inner fence is the upper adjacent value (upper horizontal green line), and the lowest value of spherical refractive error just above the lower inner fence is the lower adjacent value (below horizontal green line).

**Figure 2:**
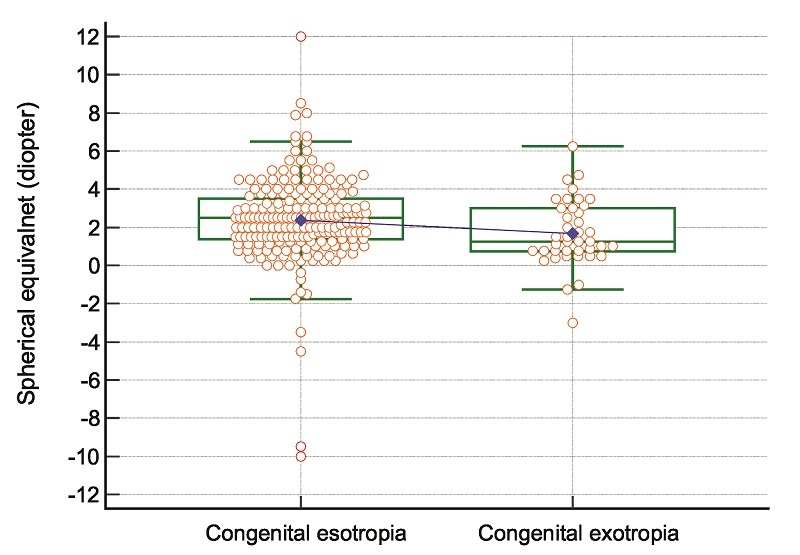
Box-and-Whisker plot for spherical equivalent refractive error in patients with congenital esotropia and congenital exotropia. The box is drawn from the 1 ${}^{st}$ to the 3 ${}^{\text{rd}}$ quartile of spherical equivalent refractive error. The horizontal line is drawn at the median of spherical equivalent refractive error, and the blue line connects the medians of the two groups. The interquartile range (IQR) is calculated as IQR = 3 ${}^{\text{rd}}$ - 1 ${}^{st}$ quartile. Also, the two imaginary lines, known as the upper and lower inner fences, are drawn at the 3 ${}^{rd}$ quartile ± 1.5 × IQR. The highest value of spherical equivalent refractive error just below the upper inner fence is the upper adjacent value (upper horizontal green line), and the lowest value of spherical equivalent refractive error just above the lower inner fence is the lower adjacent value (below horizontal green line).

**Figure 3:**
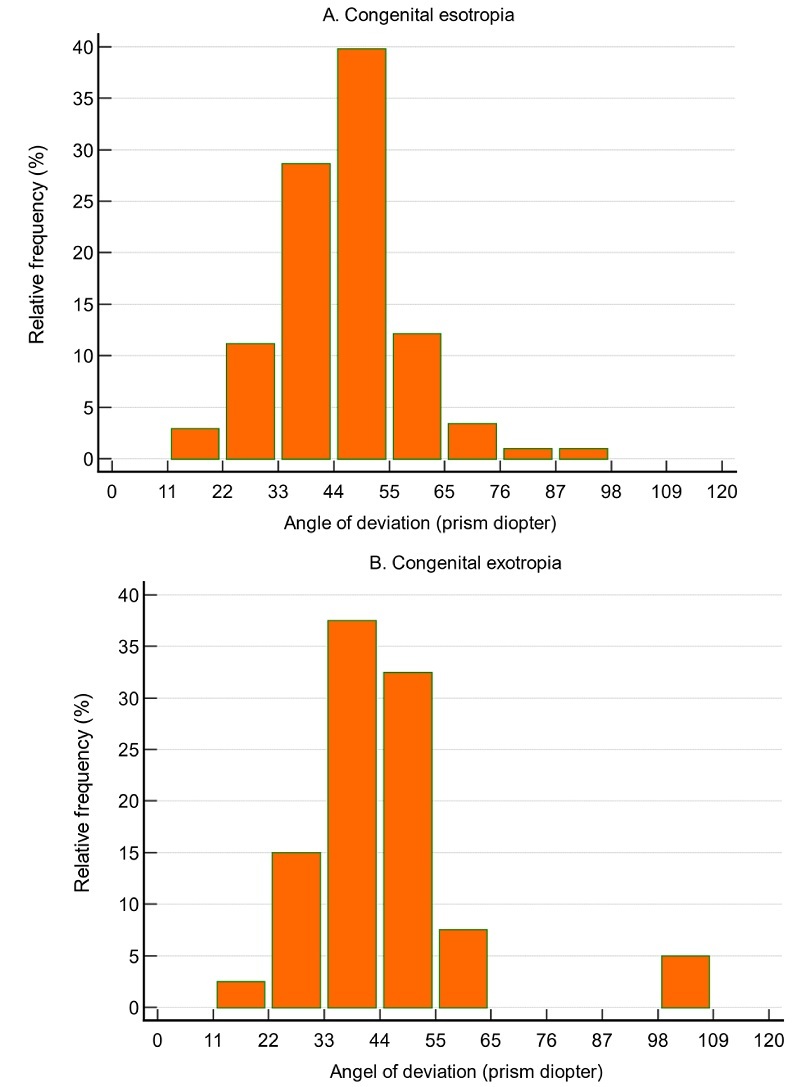
The distribution pattern of horizontal angle of deviation at near in patients with congenital esotropia (A) and congenital exotropia (B).

**Figure 4:**
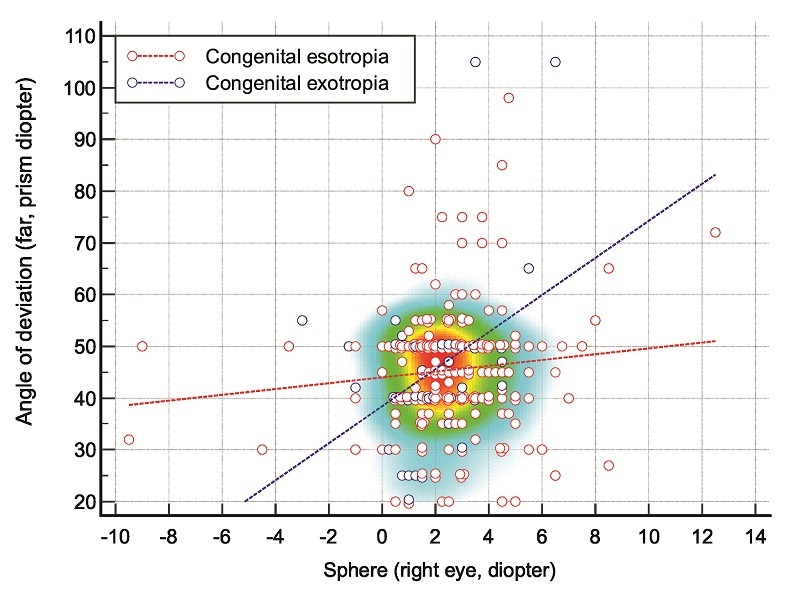
Scatter plot of spherical refractive error against angle of deviation (far, prism diopter) in patients with congenital esotropia and congenital exotropia. The red and blue dashed lines show the regression lines in congenital esotropia and congenital exotropia, respectively.

##  RESULTS

The study population included 206 patients with CET – of whom 103 (50%) were female and 103 (50%) were male – and 40 patients with CXT, comprised of 12 females (30%) and 28 males (70%) (*P *= 0.011). The age of all 246 patients at the time of examination was between 6 and 12 months, and all cases underwent surgery before they reached two years of age.

The mean sphere, cylinder, and SE in the CET group were 2.59 ± 2.28D, –0.42 ± 0.57D, and 2.38 ± 2.28D, respectively. Also, the mean horizontal angle of deviation at near was 45.5 ± 12.3 Δ (range, 20–98). Of the 206 patients with CET, 188 (91.3%) had hyperopia, 8 (3.9%) had myopia, and 10 (4.9%) were emmetropic. The most common ranges of horizontal deviation in CET were 44–55 Δ found in 40% of patients, 33–44 Δ observed in 28% of patients, and 55–65 Δ seen in 12% of patients [Figure 3A]. No patient in the CET group had vertical deviation. The distribution of spherical refractive error in patients with CET and CXT is presented in Figure 1. Figure 2 illustrates the distribution of spherical equivalent refractive error in patients with CET and CXT. The pattern of horizontal angle of deviation at near in patients with CET and CXT is shown in Figures 3A and 3B, respectively.

In the CXT group, the mean sphere, cylinder, and SE were 1.88 ± 1.84D, –0.39 ± 0.69D, and 1.69 ± 1.74D, respectively. Out of 40 patients with CXT, 35 (87.5%) had hyperopia, 3 (7.5%) had myopia, and 2 (5.0%) were emmetropic. Also, the mean horizontal angle of deviation at near was 45.0 ± 17.1 Δ (range, 20–105). In patients with CXT, the most common ranges of horizontal deviation were 33–44 Δ found in 37% of patients, 44–55 Δ seen in 32% of patients, and 22–33 Δ observed in 15% of patients [Figure 3B]. No patient in the CXT group had vertical deviation. There was a weak but statistically significant positive correlation between SE refraction and deviation angle (*r* = 0.39, *P *= 0.013).

Table 1 compares refractive error components and angle of deviation between CET and CXT. Our results show that the mean sphere component and SE in patients with CET were significantly higher than in those with CXT (both, *P *= 0.010). Patients with CXT showed a greater positive correlation between spherical refractive error and deviation angle than those with CET [Figure 4].

##  DISCUSSION

This retrospective study compared clinical features of CET and CXT in infants who underwent strabismus surgery. Significant between-group differences were found in the sphere and SE components of refractive error. These observations highlight distinct characteristics of these strabismic disorders, which are widely prevalent among pediatric patients. The mean SE refractive error was +2.38 D in the CET group and +1.69 D in the CXT group. However, the correlation between spherical refractive error and angle of deviation was stronger in patients with CXT than in those with CET. This likely reflects differing emmetropization processes in CET versus CXT, as normal infants exhibit decreasing levels of hyperopia over the first year of life.^[34]^ Disrupted emmetropization and persistent hyperopia are common in CET,^[8]^ but our data suggest that this pattern may not occur in CXT to the same degree. These results align with previous studies reporting low to moderate hyperopia within normal limits among infants with CET.^[19,20,35]^ In contrast, the refractive error distribution in CXT is thought to mirror that of the general population.^[21]^ To the best of our knowledge, this is the first direct comparison demonstrating significantly greater hyperopia in CET compared to CXT in the early infantile period.

The persistent hyperopia in CET could be related to increased accommodative convergence, which is driven by uncorrected hyperopic refractive error.^[6,36]^ Our findings highlight the need to monitor refractive development in patients with CET and those with CXT because of distinct refractive profiles.

Although CET and CXT are both categorized as large-angle strabismus, our findings showed the two conditions have similar magnitudes of horizontal angle of deviation. The mean horizontal deviation was 45.5 Δ in CET and 45.0 Δ in CXT. Furthermore, most patients with CET (40%) had horizontal deviation between 44 and 55 Δ, followed by 33–44 Δ (28%) and 55–65 Δ (12%). However, the angle of deviation in most patients with CXT (38%) ranged from 33 to 44 Δ. This contrasts with previous studies reporting larger angles of deviation in CET, often exceeding 50 Δ .^[37,38]^ However, measurement techniques and sample characteristics likely account for these discrepancies, as assessing ocular alignment in infants is challenging. Although the diagnosis of our patients was made before 6 months of age, we decided to collect data on patients between 6 and 12 months of age, as more accurate measurements could be obtained at these young ages due to better patient cooperation.

Characterizing refractive and ocular motility profiles in CET and CXT enhances helps better understand these disorders. Persistent hyperopia in CET highlights the need for regular refractive assessment and spectacle correction to reduce accommodative load. Moreover, similar horizontal alignment patterns reinforce that the magnitude of strabismus itself does not differentiate CET from CXT.

Both CET and CXT require long-term monitoring of refractive and ocular motility changes, as these factors may influence treatment planning over time. Notably, no previous study has compared different clinical characteristics, including refractive error and angle of deviation, between patients with CET and CXT. All published studies have evaluated the clinical characteristics of these patients as separate study groups. Although the nature of these two types of congenital strabismus is entirely different, this is the first study to compare refractive error and angle of deviation between CET and CXT. Our study further proves that surgery is usually necessary for both CET and CXT due to the large, infantile-onset deviations associated with these conditions.^[39]^ Younger age ( < 2 years) at surgery generally yields improved outcomes.^[40,41]^ However, postoperative drift and the need for reoperation are common, ranging from 27% to 77% in published reports.^[13,14,15]^ Preoperative refractive error and strabismus magnitude impact surgical success, with myopia and smaller angles being associated with reduced risk for reoperation.^[16,17]^ Clinical aspects highlighted include significantly greater hyperopia in patients with CET compared to CXT, emphasizing the need for regular refractive assessment.

The present study provides novel comparative data on a large sample of patients with CET and CXT. However, its retrospective cross-sectional design limited the assessment of longitudinal changes and postsurgical outcomes. Additionally, evaluating ocular alignment in infants is inherently challenging. Long-term analysis of refractive error development, ocular motility, and amblyopia in CET versus CXT will improve our understanding of how these conditions evolve over time. While the number of CXT cases over the past 10 years is relatively small, considering the relative rarity of this condition, the sample size is still reasonably large. It is important to note that all cases with neurological disorders were excluded from our study, which may have contributed to the smaller number of CXT cases.

Relating clinical profiles to treatment outcomes is also an important next step to elucidate prognostic factors. Multicenter prospective studies would allow for larger sample sizes and standardized data collection.

In summary, this study highlighted that patients with CET exhibit higher hyperopic amounts of sphere and SE than those with CXT. Although there were no significant differences in horizontal deviation between CET and CXT, most patients with CET had a greater horizontal angle of deviation than those with CXT. These differences emphasize the importance of accounting for deviation type when examining and managing patients with strabismus. While limited in scope, the present study adds to the body of literature seeking to understand these congenital disorders through detailed characterization of presenting features.

##  Financial Support and Sponsorship

None.

##  Conflicts of Interest

None.
